# The impact of sleep on complex gross‐motor adaptation in adolescents

**DOI:** 10.1111/jsr.12797

**Published:** 2018-12-18

**Authors:** Kathrin Bothe, Franziska Hirschauer, Hans‐Peter Wiesinger, Janina Edfelder, Georg Gruber, Juergen Birklbauer, Kerstin Hoedlmoser

**Affiliations:** ^1^ Laboratory for Sleep Cognition and Consciousness Research Centre for Cognitive Neuroscience University of Salzburg Salzburg Austria; ^2^ Department of Sport and Exercise Science University of Salzburg Salzburg Austria; ^3^ Department of Psychiatry and Psychotherapy Medical University of Vienna Vienna Austria

**Keywords:** adolescence, gross‐motor adaptation, motor memory consolidation, REM, sleep spindles

## Abstract

Sleep has been shown to facilitate the consolidation of newly acquired motor memories in adults. However, the role of sleep in motor memory consolidation is less clear in children and adolescents, especially concerning real‐life gross‐motor skills. Therefore, we investigated the effects of sleep and wakefulness on a complex gross‐motor adaptation task by using a bicycle with an inverse steering device. A total of 29 healthy adolescents aged between 11 and 14 years (five female) were either trained to ride an inverse steering bicycle (learning condition) or a stationary bicycle (control condition). Training took place in the morning (wake, *n* = 14) or in the evening (sleep, *n* = 15) followed by a 9‐hr retention interval and a subsequent re‐test session. Slalom cycling performance was assessed by speed (riding time) and accuracy (standard deviation of steering angle) measures. Behavioural results showed no evidence for sleep‐dependent memory consolidation. However, overnight gains in accuracy were associated with an increase in left hemispheric N2 slow sleep spindle activity from control to learning night. Furthermore, decreases in REM and tonic REM duration were related to higher overnight improvements in accuracy. Regarding speed, an increase in REM and tonic REM duration was favourable for higher overnight gains in riding time. Thus, although not yet detectable on a behavioural level, sleep seemed to play a role in the acquisition of gross‐motor skills. A promising direction for future research is to focus on the possibility of delayed performance gains in adolescent populations.

## 1 INTRODUCTION

Sleep has been shown to facilitate the consolidation of newly acquired motor memories in adults (for a review see King, Hoedlmoser, Hirschauer, Dolfen, & Albouy, 2017). However, in children and adolescents the role of sleep in motor memory consolidation is less clear. Although some studies show consolidation effects for children in speed and/or accuracy components (e.g. Astill et al., 2014), others show equal effects of sleep and wakefulness (e.g. Ashtamker & Karni, 2013) or beneficial effects of wakefulness over sleep (e.g. Wilhelm, Diekelmann, & Born, 2008). Some studies also revealed delayed gains (Desrochers, Kurdziel, & Spencer, 2016) or stronger robustness against interference after sleep (Urbain, Houyoux, Albouy, & Peigneux, 2014).

Furthermore, the effect of sleep on the consolidation of motor adaptation tasks has been debated (King et al., 2017). However, sleep‐dependent motor memory consolidation has repeatedly been linked to specific sleep features: sleep spindles and REM sleep (for a review see Diekelmann & Born, 2010). Neurofunctionally, motor adaptation is thought to rely on different cortical‐subcortical circuits during skill acquisition (Doyon, Penhune, & Ungerleider, 2003). Although the cortico‐striatal circuit seems to be important for the execution and retention of newly acquired skills during early motor adaptation learning, the cortico‐cerebellar system seems to be more relevant for long‐term retention (Doyon et al., 2003) and has been linked to rapid eye movement (REM) sleep (Smith, Nixon, & Nader, 2004). REM sleep is dominated by tonic REM phases (without eye movements) with only short interspersed episodes of phasic REM in which the characteristic rapid eye movements occur (Ermis, Krakow, & Voss, 2010; Montgomery, Sirota, & Buzsaki, 2008). Similar to slow and fast sleep spindles, it has been suggested that phasic and tonic REM might serve different functions; that is, phasic REM might facilitate greater information exchange between the hippocampus and neocortex, whereas tonic REM seems to support selective encoding and its consolidation by selective local replay and pattern separation (e.g., Bodizs et al., 2001; Hutchison & Rathore, 2015).

Smith et al. (2004) suggested that REM and N2 sleep spindles are differentially involved in the acquisition of procedural skills. According to their model, REM sleep is required when a task is new and/or skill level is poor, whereas sleep spindles are required when a task is already well learned and needs further refinement. Additionally, Fogel, Ray, Binnie, and Owen (2015) reported that REM sleep duration increased until participants mastered a procedural task. Interestingly, NREM sleep spindle density increased twice: for the gradual refinement of skills when the task was novel and for gaining further expertise when an expert level was achieved.

Moreover, data on complex gross‐motor learning are rare, but seem to indicate that increases in REM (Buchegger, Fritsch, Meier‐Koll, & Riehle, 1991) as well as NREM sleep (Morita, Ogawa, & Uchida, 2012) are favourable for overnight improvement in performance in adults. Gross‐motor tasks are complex in nature and involve a large number of skeletal muscles (Magill, 2011). In right handers, the left hemisphere plays a dominant role in the control of complex movement and skilled action (e.g. Serrien, Ivry, & Swinnen, 2006), especially when cross‐interaction with the outer environment, error processing or monitoring of response conflicts is required (e.g. Adleman et al., 2002).

The main objective of this study was to investigate whether the acquisition and consolidation of a complex gross‐motor adaptation task (i.e. riding an inverse steering bicycle (Hoedlmoser et al., 2015)) is dependent on sleep (as compared with wakefulness) in early adolescence. The inverse steering bicycle is a self‐built, conventional bike with a fixed gear ratio. The steering is constructed with two equal gear wheels so that the bicycle has to be controlled inversely by mirrored steering movements. To control for use‐dependent changes in sleep architecture (Maquet, 2001) (i.e. changes not related to the learning experience) we implemented an additional control condition in our study design (riding a stationary bicycle). Considering the high complexity of the task, we expected a novelty‐related increase in sleep spindles, most likely to be seen during N2 in the left hemisphere. Furthermore, because of the adaptive nature of the task, we assumed an involvement of REM sleep in the consolidation process. Based on previous literature, we also hypothesized that tonic and phasic REM might be differentially involved in the motor memory consolidation process.

## 2 MATERIALS AND METHODS

A total of 29 right‐handed, healthy adolescents aged between 11 and 14 years (mean [*M*] = 12.45, standard deviation [*SD*] = 0.78; five were female: sleep, 2; wake, 3) were recruited from an academic high school. Exclusion criteria included breathing difficulties, overweight (BMI above the 90th percentile of the reference population; Kromeyer‐Hauschild et al., 2001), and sleep and psychological disorders, as well as medication or drug intake that could disturb sleep or cognitive abilities. Subjects were mostly in an early pubertal stage, indicated by a mean score of 2.45 (*SD* = 0.74) on a self‐rating puberty scale (Carskadon & Acebo, 1993). For participation, subjects received shopping vouchers. Subjects and parents gave their written informed consent before study inclusion. The study was performed in accordance with the Declaration of Helsinki and approved by the local ethics committee.

### 2.1 Experimental design

**Figure 1:**
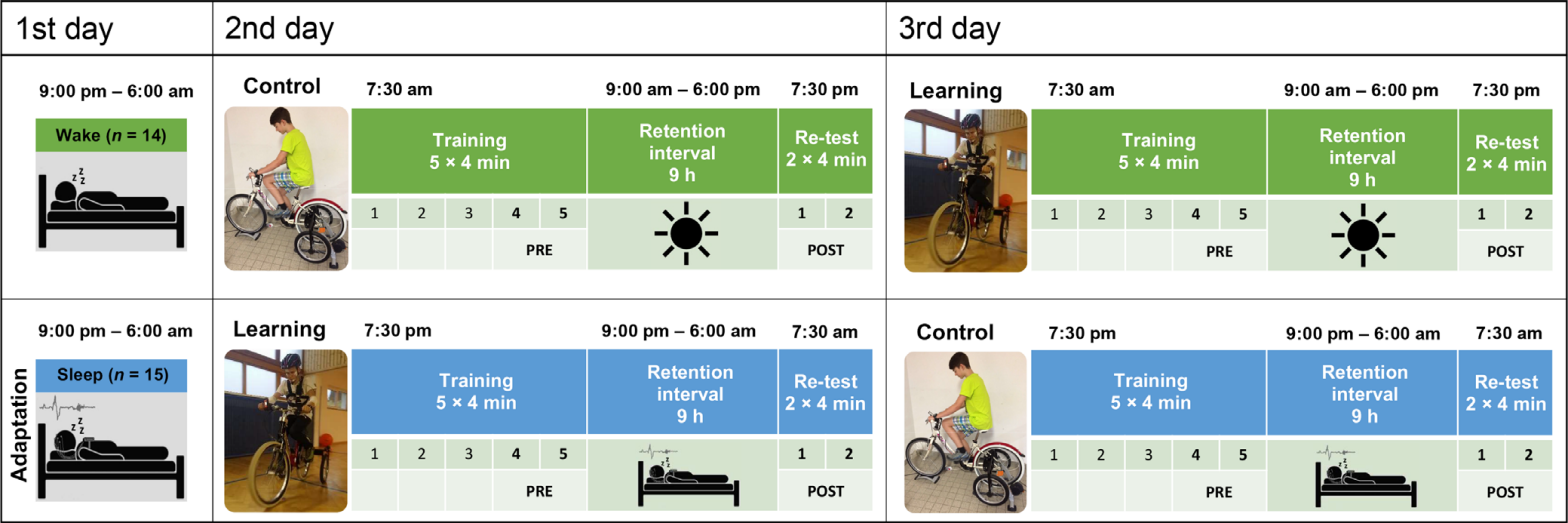
Study design. Subjects were randomly assigned to a sleep or a wake group. Within each group, participants either started with the control condition (stationary bicycle) followed by the learning condition (inverse steering bicycle with supporting wheels) or vice versa

All participants underwent an entrance examination including an evaluation of sleep habits, sport activities, cycling skills, mobile phone use, pubertal stage, intelligence and personality. Three days prior to the actual examination period, subjects started wearing wrist actigraphy (Cambridge Neurotechnologies, Cambridge, UK) and completing a sleep diary (adapted from Saletu, Wessely, Grünerger, & Schultes, 1987). All measurements, including ambulatory polysomnographic sleep recordings (PSGs), took place in familiar surroundings at the boarding school. Subjects were randomly assigned to a sleep or a wake group. As depicted in Figure 1, participants in the sleep group started with an adaptation night (21:00 to 06:00 hours, in accordance with the boarding school's sleep schedule for this age group). In the evening of day 2 (19:30 hours), they started with a training session for either the control condition (stationary bicycle) or the learning condition (inverse steering bicycle). Afterwards, they slept for 9 hr and were retested on the morning of day 3 (07:30 hours). At 19:30 hours, they trained for the respective other condition and were retested the next morning. Participants in the wake group had to follow the same protocol, except for test sessions starting in the morning and a wake retention interval of 9 hr in between. Before every training and test session, participants had to perform a psychomotor vigilance task and a tapping test (10 s of maximum speed tapping with both feet) to control for attention and fatigue. Additionally, physical effort was controlled by using a heart rate monitor (Suunto, Vantaa, Finland).

Before each session, subjects prepared for 1 min on the stationary bicycle (control condition) or performed one run along the test track without slalom cycling (learning condition, cf. Figure 1). In the control condition, subjects were asked to ride the stationary bicycle with constant speed at 20 W per kg bodyweight and a cadence between 50 and 60 rotations per minute. In the learning condition, subjects were asked to ride the inverse steering bicycle through a partially irregular slalom course (25 m) with eight turning points (2.5 m apart) as quickly and as accurately as possible. The turning points were marked with rubber disks and had to be run over with the front wheel of the bicycle. Participants were informed that missing a marker (riding error) would result in a time penalty of 2 s. For the test, participants performed five 4‐min blocks with 1 min rest in between each block. The retest consisted of two 4‐min blocks. After training, participants filled out a questionnaire evaluating motivation during training. To cope with the task, the inverse steering bicycle was equipped with supporting wheels and all subjects wore a helmet for safety reasons. A rotatory potentiometer mounted in the head tube of the inverse bike was used to assess riding performance by the standard deviation of the steering angle (SDSA [°]). In addition to steering accuracy, the mean riding time (s) was measured and adjusted for riding errors per block.

### 2.2 Polysomnography

In the sleep group, PSG was recorded using an ambulatory 16‐channel amplifier (Varioport©, Becker Meditec, Karlsruhe, Germany) during adaptation, control and learning nights. PSG started at 21:00 hours and was terminated after 9 hr of time in bed (06:00 hours). Data were recorded referentially against a common reference at Cz and re‐referenced offline to contralateral mastoids (A1, A2). PSG recordings included 10 electroencephalogram (EEG) channels (F3, Fz, F4, C3, C4, P3, Pz, P4, O1 and O2), two horizontal electrooculogram (EOG) channels, two vertical EOG and two chin electromyogram (EMG) channels and were obtained at a sampling rate of 512 Hz. Sleep was automatically staged (Somnolyzer 24.9.7; Koninklijke Philips N.V., Eindhoven, the Netherlands) and visually controlled by an expert scorer according to the American Academy of sleep Medicine criteria (Iber, Ancoli‐Israel, Chesson, & Quan, 2007). Sleep spindles during N2 were detected automatically for electrode positions F3, F4, C3 and C4. The automatic spindle detection (ASK analyzer, The Siesta Group, Vienna, Austria) was twofold. In the first step, “possible” spindle events were detected by the band‐pass method (Schimicek, Zeitlhofer, Anderer, & Saletu, 1994) with the following criteria: (a) band‐pass filter 11–16 Hz, (b) amplitude > 12 μV and (c) duration 300–2000 ms. In the second step, the “possible” spindle events detected by this low specificity/high sensitivity method were further evaluated in order to increase specificity. From all “possible” spindle episodes, “certain” spindle episodes were identified by linear discriminate analysis (LDA) trained on visually scored spindles. The LDA uses the five log‐transformed features (spindle duration and mean amplitudes in four frequency bands: spindle, theta, alpha and fast beta) of “possible” spindles. For all our analyses, we only used spindle events with a discriminant score > 1.7. This corresponds to a specificity of 98%, which is similar to visual scorers (for more details, see Anderer et al., 2005). Furthermore, we visually inspected the spindles identified by the algorithm to ensure valid detections. To evidence the distribution of sleep spindle peaks, we calculated EEG power spectral density for frontal (F3 and F4) and central (C3 and C4) leads during N2 sleep (for details see Hoedlmoser et al., 2014). Sleep spindle peak frequency was defined as the maximal deflection between 10 and 16 Hz and was semi‐automatically detected for each subject. The average peak frequency was restricted to the slow sleep spindle range between 11 and 13 Hz (F3, range = 9.50–12.75, *M* = 11.88, *SD* = 0.61; F4, range = 9.00–13.00, *M* = 11.90, *SD* = 0.81; C3, range = 9.50–13.00, *M* = 12.51, *SD* = 0.53; C4, range = 9.50–13.25, *M* = 12.51, *SD* = 0.54; cf. Supporting Information Figure S1). No additional fast sleep spindle peak was evidenced between 13 and 15 Hz. Because our analyses did not yield evidence for two distinct spindle peaks, all further analyses were solely focused on slow SpA during N2 sleep. Spindle activity (SpA) was estimated by using an algorithm that gives an integer value for the envelope spanning the respective wave complexes within a 30‐s epoch; that is, it captures the duration as well as the amplitude of identified spindles and thus reflects the power or intensity of the spindle process (SpA = mean spindle duration * mean spindle amplitude; Schabus et al., 2004; Hahn et al., 2018). Because it has been suggested that spindles are involved in synaptic plasticity and long‐term potentiation processes (e.g., Rosanova & Ulrich, 2005), with spindle amplitudes being important for the extent of hippocampal‐neocortical memory reactivation (e.g., Bergmann, Mölle, Diedrichs, Born, & Siebner, 2012) and spindle duration being important for the optimal timing of information transfer into the cortex (e.g., Bonjean et al., 2011), SpA might be a sensible measure for reflecting state‐like or learning‐dependent consolidation processes (Lustenberger, Wehrle, Tüshaus, Achermann, & Huber, 2015). Furthermore, for REM sleep periods we differentiated between tonic and phasic REM intervals. The duration of every type was then accumulated to get a total night time value (min) of tonic and phasic REM duration.

### 2.3 Statistical analyses

Statistical analyses were performed using IBM SPSS Statistics 24 (IBM, Armonk, NY, USA). The significance level was set to *p* < 0.05. Effect sizes are provided as eta squared (η^2^).

To examine whether subjects of both groups (sleep and wake) showed a learning effect from training block one to five, dependent samples *t* tests were conducted for riding accuracy (SDSA) and riding time. For investigating the effects of sleep and wake retention intervals on change in performance, mean performance values for SDSA and riding time from training blocks 4 and 5 (pre) and from retrieval blocks 1 and 2 (post) were calculated (cf. Table 1). Subsequently, we conducted two‐factor analyses of variance (ANOVA) for repeated measures with the within‐subject factor time (pre vs. post) and the between‐subject factor group (sleep vs. wake) for SDSA and riding time. In a next step, we examined whether overnight changes in SDSA and riding time were associated with changes in sleep parameters. Subjects were divided into two groups: participants who enhanced their N2 slow SpA in the left or right hemisphere from control to learning night and participants with no SpA‐enhancement. SpA‐changes were computed as learning minus control night (i.e. positive values represent an increase in SpA from control to learning night) using 0 as the cut‐off score (cf. Table 3). Note that we had to exclude one participant because of artifacts in the spindle analyses. Similarly, enhancement and non‐enhancement groups have been formed for REM (total night, tonic and phasic, cf. Table 3). To test whether enhancement groups differed in absolute change in performance, two‐way ANOVAs were computed for SDSA and riding time with the within‐subject factor time (pre vs. post) and the between‐subjects factor enhancement (enhancers vs. non‐enhancers). Pearson correlations (two‐tailed) were used to test whether overnight changes in gross‐motor performance linearly relate to N2 slow SpA and REM duration during learning night and control night, as well as to changes between learning and control night.

**Table 1:** Descriptive data for behavioural measures

	Sleep	Wake	*t*	*p*	η^2^
Behavioural measures					
SDSA (°)					
Training (change from block 1 to block 5)	−2.76 ± 3.65	−2.49 ± 2.83	−0.216	0.831	0.001
Pre (last 2 training blocks)	21.40 ± 1.89	21.89 ± 2.60	−0.588	0.561	0.013
Post (2 retrieval blocks)	21.37 ± 2.62	21.82 ± 2.23	−0.497	0.623	0.009
Performance change (post‐pre)	−0.02 ± 1.61	−0.07 ± 1.39	0.077	0.939	<0.001
Riding time (s)					
Training (change from block 1 to block 5)	−12.03 ± 6.30	−15.09 ± 5.70	1.365	0.184	0.065
Pre (last 2 training blocks)	19.35 ± 2.50	20.41 ± 2.24	−1.204	0.239	0.051
Post (2 retrieval blocks)	16.91 ± 1.87	17.98 ± 2.20	−1.418	0.168	0.069
Performance change (post‐pre)	−2.44 ± 1.65	−2.43 ± 0.96	−0.021	0.983	<0.001

SDSA, standard deviation of the steering angle.

## 3 RESULTS

### 3.1 Behavioural data

Performance in both groups improved significantly over training (blocks 1–5), indicating that subjects became better at riding the inverse steering bicycle (sleep: SDSA, *t*_14_ = 2.927, *p* = 0.011, η^2^ = 0.380; riding time, *t*_14_ = 7.399, *p* < 0.001, η^2^ = 0.796; wake: SDSA, *t*_13_ = 3.296, *p* = 0.006, η^2^ = 0.455; riding time, *t*_13_ = 9.896, *p* < 0.001, η^2^ = 0.883; for details on the learning curves please refer to Supporting Information Figures S2 and S3) (Table 1).

**Figure 2:**
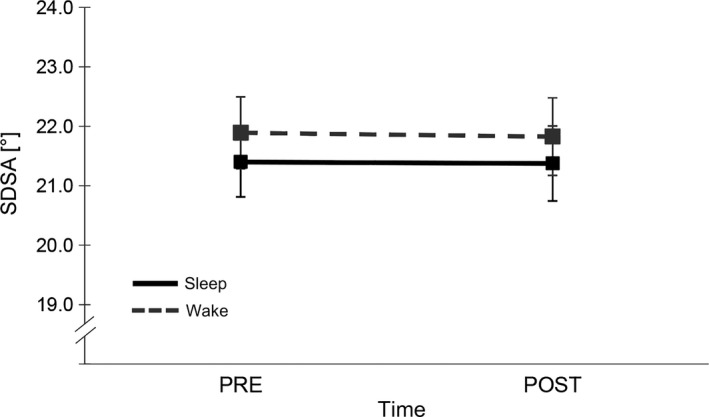
Standard deviation of the steering angle (SDSA) in the sleep and wake groups. Stabilization of SDSA over both retention intervals and no group differences. High SDSA values indicate low performance. Error bars represent standard error of the mean

No significant main (group, *F*_1,27_ = 0.326, *p* = 0.573, η^2^ = 0.012) or interaction (group × time, *F*_1,27_ = 0.006, *p* = 0.939, η^2^ < 0.001) effect could be found for SDSA, indicating that the sleep and wake groups showed similar task accuracy throughout training and retrieval regardless of spending the retention interval asleep or awake (cf. Figure 2). Furthermore, data showed that riding accuracy was stabilized after both retention intervals (time, *F*_1,27_ = 0.024, *p* = 0.877, η^2^ = 0.001).

**Figure 3:**
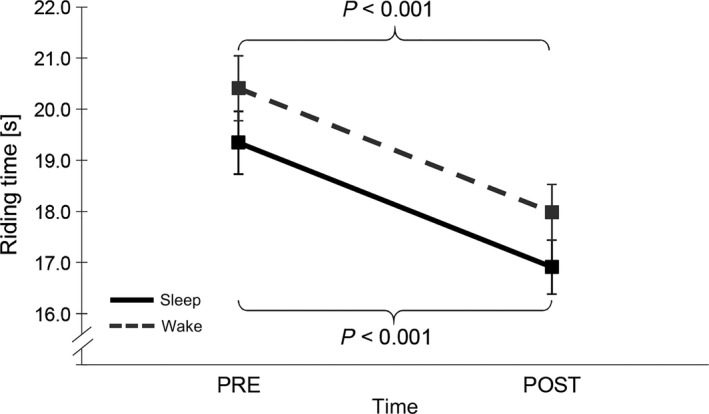
Riding time in the sleep and wake groups. Both the sleep and wake groups significantly reduce their riding time over the retention interval. High riding time values indicate low performance. Error bars represent standard error of the mean

Additionally, subjects in both groups showed a significant decrease in riding time after the retention interval (time, *F*_1,27_ = 92.438, *p* < 0.001, η^2^ = 0.774; cf. Figure 3).

### 3.2 Sleep data

Dependent sample *t* tests revealed no significant differences in sleep architecture between the learning and control nights (cf. Tables 2 and 3).

**Table 2:** Sleep architecture

	Adaptation	Learning	Control	Learning versus Control		
				*t*	*p*	η^2^
Sleep architecture						
Total sleep time (TST)	510.20 ± 11.29	503.90 ± 18.42	499.03 ± 18.39	0.777	0.450	0.041
Sleep onset latency (SOL)	18.10 ± 10.20	17.33 ± 12.64	15.23 ± 9.33	0.562	0.583	0.022
Sleep efficiency (EFF)	96.80 ± 1.06	97.24 ± 2.34	97.10 ± 2.11	0.194	0.849	0.002
Wake after sleep onset (WASO)	6.15 ± 1.59	6.53 ± 4.43	7.90 ± 6.77	−0.712	0.488	0.035
N1, %	39.45 ± 6.22	5.72 ± 2.52	6.33 ± 1.59	−1.026	0.322	0.070
N2, %	31.04 ± 5.84	40.68 ± 8.10	39.41 ± 6.87	0.709	0.490	0.035
N3, %	23.36 ± 3.45	31.48 ± 6.41	32.53 ± 6.90	−0.842	0.414	0.048
REM, %	7.37 ± 4.52	22.12 ± 7.83	21.73 ± 3.41	0.168	0.869	0.002
Spindle activity (SpA)						
Hemisphere						
N2 left	19.68 ± 2.61	19.36 ± 2.91	19.60 ± 3.12	−1.132	0.278	0.090
N2 right	19.61 ± 3.10	19.30 ± 3.17	19.26 ± 1.98	0.069	0.946	<0.001
REM duration measures						
REM	128.13 ± 20.63	118.07 ± 46.76	118.23 ± 22.18	−0.012	0.990	<0.001
Tonic REM	59.33 ± 14.99	57.60 ± 26.77	56.73 ± 23.75	0.111	0.913	<0.001
Phasic REM	59.77 ± 12.06	54.17 ± 23.75	51.57 ± 12.48	0.443	0.665	0.014

**Table 3:** Descriptive data for SpA/REM enhancers and non‐enhancers

	Enhancers							Non‐enhancers						
	Learning	Control	*N*	*t*	*p*	η^2^	Change	Learning	Control	*N*	*t*	*p*	η^2^	Change
Spindle activity (SpA)														
Hemisphere														
N2 left	20.07 ± 3.10	19.69 ± 3.16	7	3.087	0.021	0.544	0.38 ± 0.33	18.65 ± 2.74	19.52 ± 3.32	7	−3.670	0.010	0.627	−0.87 ± 0.62
N2 right	20.00 ± 3.56	19.43 ± 3.44	8	4.011	0.005	0.641	0.57 ± 0.40	18.37 ± 2.56	19.48 ± 3.11	6	−4.109	0.009	0.707	−1.11 ± 0.66
REM duration measures														
REM	149.93 ± 29.55	110.86 ± 17.34	7	4.867	0.003	0.772	39.07 ± 21.24	90.19 ± 41.49	124.69 ± 24.99	7	−2.111	0.073	0.389	−34.50 ± 46.23
Tonic REM	70.61 ± 23.85	50.17 ± 12.67	9	3.800	0.005	0.616	20.44 ± 16.14	38.08 ± 18.28	66.58 ± 12.08	5	−3.454	0.018	0.705	−28.50 ± 20.21
Phasic REM	63.65 ± 17.97	50.45 ± 12.65	10	3.522	0.006	0.554	13.20 ± 11.85	35.20 ± 23.94	53.80 ± 13.28	4	−1.631	0.178	0.399	18.60 ± 25.50

### 3.3 Sleep spindle activity

**Figure 4:**
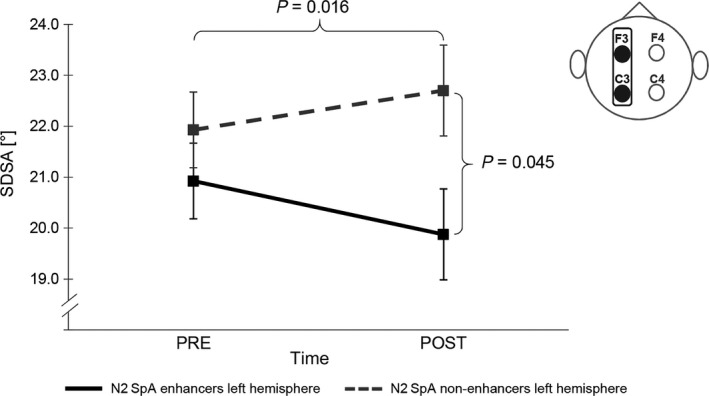
Pre‐ and post‐performance differences in standard deviation of the steering angle (SDSA) for left hemisphere N2 slow SpA enhancers and non‐enhancers. A decrease in left hemispheric N2 slow SpA from the control to the learning night leads to a significant overnight decrease in SDSA, whereas a slow SpA increase seems to stabilize SDSA. High SDSA values indicate low steering accuracy. Error bars represent standard error of the mean

For SDSA, the results (cf. Figure 4) showed a specific left hemispheric interaction between time and enhancement (*F*_1,12_ = 6.349, *p* = 0.027, η^2^ = 0.343), indicating that an increase in N2 slow SpA from the control to the learning night tended to stabilize SDSA levels (*t*_6_ = 1.532, *p* = 0.176, η^2^ = 0.281), whereas a decrease in N2 slow SpA led to a decrease in SDSA after sleep (*t*_6_ = −3.330, *p* = 0.016, η^2^ = 0.649).

**Figure 5:**
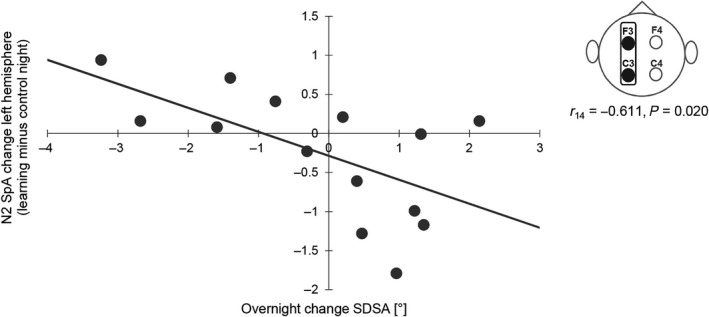
Standard deviation of the steering angle (SDSA) and left hemispheric N2 slow SpA. The greater the improvement in SDSA overnight, the higher the increase in N2 slow SpA from the control to the learning night. Negative SDSA values indicate improvement in performance overnight; a positive change in SpA values indicates an increase in SpA from the control to the learning night. Error bars represent standard error of the mean

Pearson correlations showed that specifically the change in left hemispheric N2 slow SpA from the control to the learning night was related to overnight improvement in SDSA (cf. Figure 5).

For riding time, results showed an overall decrease in riding time with sleep for both N2 SpA enhancers and non‐enhancers for both hemispheres. However, Pearson correlations did not reveal significant results, indicating that SpA did not have an influence on changes in riding time.

### 3.4 REM sleep

Results for SDSA and REM revealed a main effect for the factor enhancement (*F*_1,13_ = 6.522, *p* = 0.024, η^2^ = 0.502). Post hoc independent sample *t* tests indicated that an enhancement in REM duration was associated with worse SDSA compared with participants with no REM enhancement (pre_REM_, *t*_9.523_ = 1.860, *p* = 0.094, η^2^ = 0.217; post_REM_, *t*_13_ = 2.729, *p* = 0.017, η^2^ = 0.364). Pearson correlations showed that a decrease in REM from the control to the learning night (*r*_15_ = 0.581, *p* = 0.023), as well as less time spent in REM during the learning night (*r*_15_ = 0.527, *p* = 0.044), was associated with higher overnight improvement in steering accuracy. However, we did not find a significant correlation for the control night (*r*_15_ = −0.252, *p* = 0.366), indicating that REM changes occurred as a result of learning our gross‐motor adaptation task.

**Figure 6:**
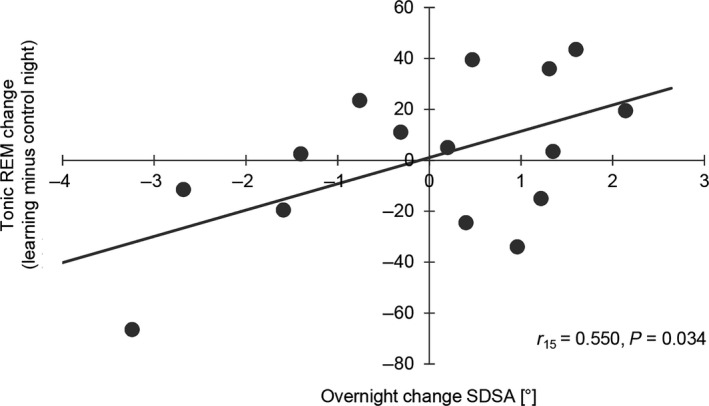
Standard deviation of the steering angle (SDSA) and duration of tonic rapid eye movement sleep (REM). A decrease in duration of tonic REM from the control to the learning night leads to a higher overnight improvement in steering accuracy. Negative overnight change values indicate an improvement in performance. Error bars represent standard error of the mean

Although a comparison between tonic REM enhancers and non‐enhancers failed to reach significance (enhancement: *F*_1,13_ = 3.046; *p* = 0.105; η^2^ = 0.234), Pearson correlations indicated that overnight improvement in SDSA was associated with a decrease in tonic REM duration from the control to the learning night (cf. Figure 6), as well as with less time spent in tonic REM during the learning night (*r*_15_ = 0.726, *p* = 0.002). There was no correlation for the control night (*r*_15_ = 0.196, *p* = 0.485).

Concerning riding time and REM sleep, both REM duration enhancers and non‐enhancers were able to improve their riding times overnight (time, *F*_1,13_ = 41.161, *p* < 0.001, η^2^ = 0.709), with a tendency for greater improvements in REM duration enhancers (time × enhancement, *F*_1,13_ = 3.931, *p* = 0.069, η^2^ = 0.068). Pearson correlations neither revealed significant relations between overnight changes in speed and REM durations from the control to the learning night (*r*_15_ = −0.358, *p* = 0.190) nor between changes in speed and REM duration during the learning night (*r*_15_ = −0.359, *p* = 0.189).

**Figure 7:**
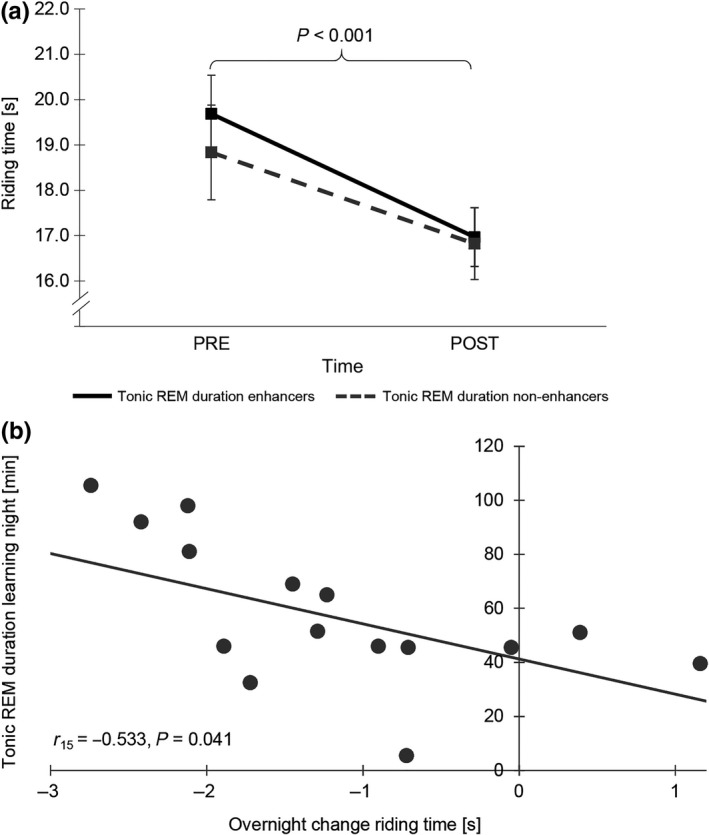
(a) Riding time and duration of tonic rapid eye movement sleep (REM). Tonic REM duration enhancers show a more significant overnight improvement in riding time compared with non‐enhancers. (b) A higher overnight improvement in riding time is associated with more time spent in tonic REM during the learning night. High riding time values indicate low speed. Error bars represent standard error of the mean

For tonic REM, however, additionally to a main effect for time (*F*_1,13_ = 28.931, *p* < 0.001, η^2^ = 0.679; cf. Figure 7), a Pearson correlation indicating that longer tonic REM durations during the learning night were favourable for more overnight improvement in riding time (cf. Figure 7) has been found. Although the correlation for tonic REM changes from the control to the learning night failed to reach significance (*r*_15_ = −0.435, *p* = 0.106), there was also no correlation with speed for the control night (*r*_15_ = −0.079, *p* = 0.779), thus suggesting that changes in tonic REM occurred as a result of gross‐motor adaptation learning.

## 4 DISCUSSION

In this study, we examined sleep‐dependent motor memory consolidation in adolescents by using a gross‐motor adaptation task, that is, riding an inverse steering bicycle. On a behavioural level, we could not find evidence for sleep‐dependent memory consolidation. Instead, similar contributions of sleep and wake to motor memory consolidation have been observed. This has been found in other studies as well (e.g. Ashtamker & Karni, 2013). However, analyzing our sleep data, we found N2 slow SpA and REM duration changes between the control and learning nights to be associated with overnight changes in performance, thus hinting at the possibility of covert sleep‐dependent changes and delayed performance gains. Hence, sleep might provide the environment for a more deeply integrated and consolidated memory trace.

Regarding the similar behavioural outcomes in both the sleep and wake groups, two possible mechanisms might have played a role. (a) The nature of the task: we consider riding an inverse steering bicycle as a mainly implicit task because it is constrained to fast corrective movements in order to avoid falling (Curran, 2001). According to Robertson, Pascual‐Leone, and Press (2004) and Robertson (2009), offline learning of implicit tasks has been observed regardless of whether the retention interval contained sleep or not. Thus, observing similar changes in both the sleep and wake groups seems to be plausible. Although implicit learning is considered to maintain or even improve without sleep or further practice, it has been shown that sleep can make important contributions to unintentionally acquired skills and memories. For example, a study by Peigneux et al. (2003) showed learning‐dependent reactivation of implicit, non‐declarative memories (probabilistic serial reaction time task) during REM sleep. Nevertheless, riding an inverse steering bicycle also involves explicit and thus sleep‐dependent components: (i) aiming to achieve the goal of riding the inverse steering bicycle (goal component), (ii) being aware that the task involves acquiring a new skill, and (iii) recognizing improvements in performance during training (Robertson, 2009; Robertson et al., 2004). (b) The developmental stage and task difficulty/pre‐retention interval skill level: according to Adi‐Japha and Karni (2016), before puberty consolidation processes are reflected in enhancement of task performance over sleep and wake periods equally. Considering that our sample was on average in an early pubertal stage, this notion might apply to our data as well. Furthermore, competing memory systems during wakefulness and sleep may have led to similar outcomes in both groups. In adults, motor skill learning can block the consolidation of declarative components during wakefulness but not during sleep, because declarative and motor systems operate independently during this stage (Robertson, 2009). However, this might be different in children and adolescents for developmental reasons. We assume that participants did not reach asymptotic performance at the end of training. As in other complex gross‐motor tasks (e.g. skiing), no ceiling effects could be expected after our relatively short period of training because these kinds of skills can be improved even after years of experience as a result of the vast amount of degrees of freedom involved (Wulf & Shea, 2002). Thus, although both the sleep and wake groups showed a significant improvement in performance over the training interval, their skill level might have still been too low to significantly benefit from sleep. In fact, this has been shown in earlier adult (e.g. Hauptmann, Reinhart, Brandt, & Karni, 2005; Karni & Sagi, 1993; Korman et al., 2007) studies and might have been the case in previous children/adolescent studies as well (Fischer, Wilhelm, & Born, 2007; Prehn‐Kristensen et al., 2009; Urbain et al., 2014). According to Wilhelm, Metzkow‐Meszaros, Knapp, and Born (2012), children's implicit skill memories reach a level of strength and independence only after extended training. With only weak implicit skill representations (i.e. no sufficient pre‐retention skill level) there might have been interactions between implicit and explicit motor systems during offline sleep consolidation (Albouy, King, Maquet, & Doyon, 2013; Wilhelm et al., 2012). Thus, any sleep‐dependent behavioural gains in implicit motor performance may have been nullified by competing interactions with explicit aspects that are primarily strengthened during sleep; that is, motor systems are not yet operating independently during sleep for developmental reasons. According to Stickgold (2009), an intermediate skill level is best for sleep‐dependent improvements in performance. Compared with adults, motor performance is slower and less automatized in children and adolescents. This coincides with less hippocampal activation during motor learning and might thus be insufficient for sleep‐related benefits (Wilhelm et al., 2012). Taking this into account, the absence of differences between the sleep and wake groups at the behavioural level might have been caused by a similar interference of memory systems during sleep and wake retention intervals.

However, the results indicated that improvement in SDSA was related to a left hemispheric N2 slow SpA increase as well as to a decrease in REM duration, whereas improvements in riding time seemed to be more related to an increase in REM sleep duration, with an emphasis on time spent in tonic REM.

Sleep spindles have repeatedly been linked to procedural memory consolidation (for a review see Fogel & Smith, 2011). Most studies reported an increase in fast spindle parameters being important for overnight increases in performance. Nevertheless, in some cases slow spindles have also been related to sleep‐dependent motor memory consolidation processes (e.g. Astill et al., 2014). Taking the developmental aspect into account, it has been shown that, until the end of puberty, slow spindles are more dominant than fast spindles across the scalp. In fact, spindle data from our sample fit our earlier findings (Hahn et al., 2018; Hoedlmoser et al., 2014), with predominant peaks in the slow (11–13 Hz) but not in the fast (13–15 Hz) sleep spindle frequency range (cf. Supporting Information Figure S1). Therefore, it seems to be possible that our slow SpA results partly reflect this developmental characteristic. Considering the lateralized nature of our spindle effect, it has previously been stated that the left hemisphere plays a dominant role in the control of complex movements and skilled action (e.g. Serrien et al., 2006). Moreover, left prefrontal and fronto‐central regions are involved in error processing, monitoring of response conflicts, and response inhibition (e.g. Adleman et al., 2002). Thus, a left hemispheric involvement in establishing and refining the skill of riding an inverse steering bicycle, while simultaneously inhibiting the highly automated movement pattern of riding a normal bicycle, seems to be plausible. Furthermore, slow spindles have been associated with cortico‐cortical activity and are considered to be important for the formation of long‐term memory representations (Mölle, Bergmann, Marshall, & Born, 2011). It has been suggested that with skill development, motor representations that become established in the left hemisphere are supporting cortico‐cortical information processing for optimal task performance (Serrien et al., 2006). Thus, an increase in left hemispheric slow SpA after learning motor adaptation could reflect continued task optimization processes during sleep. Moreover, a study by Svatkova et al. (2015) reported a left hemispheric increase in white matter integrity for right‐handed participants after 6 months of training on a stationary bicycle. Connectivity increases were located in fibre bundles relevant to adaptation to changing motor demands. These results might hint at the underlying gross‐motor network involved during bicycling. Thus, our spindle results might reflect offline reactivations of this “bicycle network” for long‐term motor memory consolidation and adaptation purposes.

In the present study, an increase in REM was associated with improvements in riding time. REM has been linked to consolidation processes occurring within the cortico‐cerebellar motor system during motor adaptation (Doyon et al., 2003; Smith et al., 2004). It is thought to be part of an implicit motor control component that regulates and optimizes movement dynamics such as speed, timing and sensorimotor integration (Penhune & Steele, 2012). The cortico‐cerebellar system is also involved in the formation and long‐term storage of internal models of newly acquired skills (Penhune & Steele, 2012). Thus, an improvement in the speed component with an associated increase in REM sleep might reflect cortico‐cerebellar network activity for task optimization in terms of movement dynamics. Moreover, tonic REM has been suggested to support memory consolidation by selective local replay of newly acquired memories and reduction of interference through pattern separation (e.g. Hutchison & Rathore, 2015). Therefore, an increase in tonic REM might have been beneficial for separating the skill of riding a normal bicycle from riding an inverse steering bicycle, hence resulting in an overnight improvement in speed.

Considering the fact that an increase in REM seemed to facilitate speed whereas a decrease in REM and an increase in SpA seemed to facilitate accuracy, it is tempting to assume that our task involved two networks that have previously been described for sequential motor learning (Albouy et al., 2013; Hikosaka, Nakamura, Sakai, & Nakahara, 2002): a spatial network (for improvements in accuracy) supported by parietal‐prefrontal cortical loops, including the hippocampus, and a motor network supported by motor‐cortical loops (for improvements in speed), including the striatum and the cerebellum. Accordingly, the increase in SpA associated with performance gains in the accuracy component suggests hippocampus‐dependent memory consolidation processes, whereas the increase in REM for gains in speed suggests a more implicit striato‐cortical or cerebello‐cortical consolidation (Peigneux et al., 2003). Thus, it might be possible that our results reflect two networks that by interaction improve different task components during alternating sleep stages. This interpretation would support the sequential hypotheses idea, which proposes that it is the repetitive cyclic sequence of NREM to REM that facilitates learning during sleep (Giuditta, 2014). Regarding task accuracy, not only an increase in slow SpA, but also a decrease in REM seemed to be favourable for improvements in performance. This result fits with the model of Smith et al. (2004) and the work of Fogel et al. (2015) suggesting that REM is only involved when a skill is particularly novel and has not yet been mastered. Thus, subjects with a decrease in REM might have already reached a higher skill level, possibly allowing for more refined processing during NREM sleep. However, there was no statistically significant correlation between a decrease in REM and a parallel increase in SpA in our data.

Lastly, it has to be noted that our sample had a bias towards male participants. Given that male and female subjects show differences in brain maturation from childhood to adolescence, the sample composition has to be considered as a limitation of our study. However, statistical tests for group differences did not reveal any relevant behavioural or sleep‐related differences between female and male participants (cf. Supporting Information Table S1–S3).

In summary, our results suggest that sleep played a covert role in the acquisition of our gross‐motor adaptation task. Although there were no behavioural differences between the sleep and wake groups after the retention interval, data indicate that slow SpA and duration of REM changed as a result of learning to ride an inverse steering bicycle. We cannot exclude that sleep affected behavioural performance later on or that benefits of sleep over wakefulness would have come to light when tested against interference. On account of this, further research in adolescents may focus on the possibility of delayed performance gains or robustness against memory interference.

## CONFLICT OF INTEREST

This was not an industry‐supported study. None of the authors has any financial conflict of interest.

## AUTHOR CONTRIBUTIONS

K.B. collected data, analysed the data, performed the statistical analysis, interpreted the results and drafted the manuscript. F.H. designed the study and collected data. W.H‐P. and E.J. collected and analysed the data. G.G. analysed the data. K.H. and J. B. designed the study, interpreted the results and drafted the manuscript. J. B. custom built the inverse steering bicycle. All authors read and approved the final manuscript.
