# Supplementary material for: The impact of sleep on complex gross‐motor adaptation in adolescents
Source: J Sleep Res. 2018 Dec 18;28(4):e12797. doi: 10.1111/jsr.12797 (PMC6766860; doi:10.1111/jsr.12797)
Supplement: Supplementary file 1 [file JSR-28-na-s001.docx]

**SUPPORTING INFORMATION**

**The impact of sleep on complex gross-motor adaptation in adolescents**

Bothe, K.^1^, Hirschauer, F. ^1^, Wiesinger, H-.P. ^2^, Edfelder, J. ^2^, Gruber, G. ^3^, Birklbauer, J. ^2*^, & Hoedlmoser, K.^1*^

**^1^**Laboratory for Sleep, Cognition and Consciousness Research, Centre for Cognitive Neuroscience, University of Salzburg, Salzburg, Austria

^2^Department of Sport Science and Kinesiology, University of Salzburg, Salzburg, Austria

^3^Department of Psychiatry and Psychotherapy, Medical University of Vienna, Vienna, Austria.

*Corresponding author information:

Kerstin Hoedlmoser, University of Salzburg,

Centre for Cognitive Neuroscience, Laboratory

for Sleep, Cognition and Consciousness

Research, Hellbrunnerstr.34,

5020 Salzburg, Austria.

Tel.: +43-662-8044-5143;

e-mail: kerstin.hoedlmoser@sbg.ac.at

**Supporting Information Figures**


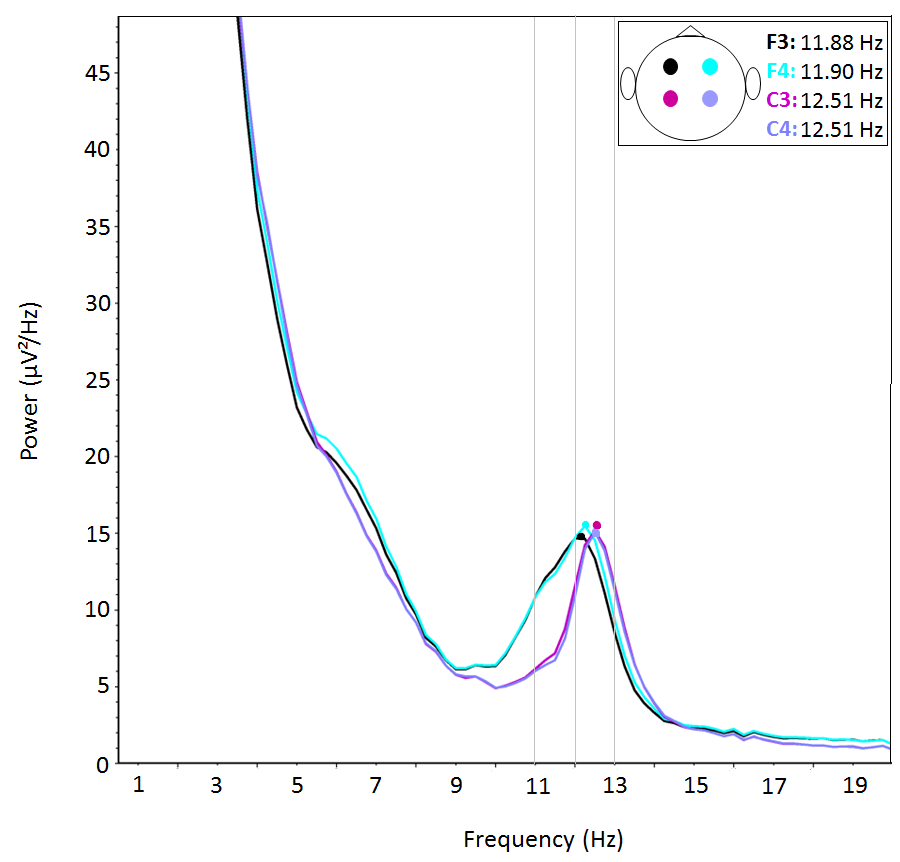


**Figure 1.** Electroencephalographic (EEG) power spectral density for frontal (F3, F4) and central (C3, C4) leads during N2 sleep. Mean power spectral density over all subjects and all nights (adaptation, learning, control). Sleep spindle peak frequency is defined as the maximal deflection between 10-16 Hz and was detected semiautomatically for all four electrode sites. Note that the average sleep spindle peak frequency of all electrode sites is below 13 Hz, and thus restricted to the slow spindle frequency range.


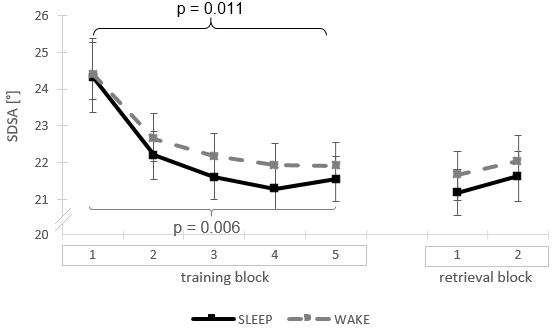


**Figure 2.** Learning curve for accuracy (SDSA) across training and retrieval blocks.


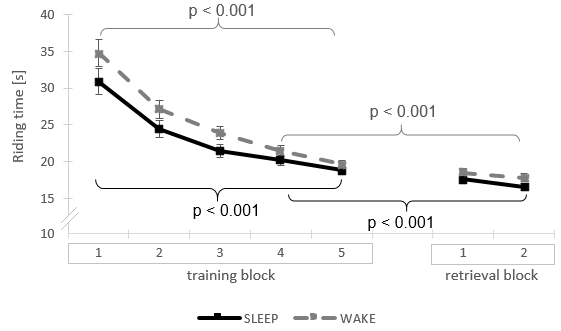


**Figure 3.** Learning curve for speed (riding time) across training and retrieval blocks.

**Supporting Information Tables**

**Table 1.** Behavioral measures male (n=24) vs. female (n=5) overall.

| BEHAVIORAL MEASURES | | | | | |
| --- | --- | --- | --- | --- | --- |
|  | male | female | t | P | η^2^ |
| SDSA [°] |  |  |  |  |  |
| PRE (last 2 training blocks) | 21.42 ± 2.18 | 22.65 ± 2.47 | -1.120 | 0.273 | 0.044 |
| POST (2 retrieval blocks) | 21.28 ± 2.39 | 23.08 ± 2.09 | -1.564 | 0.129 | 0.083 |
| Riding time [s] |  |  |  |  |  |
| PRE (last 2 training blocks) | 19.60 ± 2.49 | 21.08 ± 1.56 | -1.269 | 0.215 | 0.056 |
| POST (2 retrieval blocks) | 17.11 ± 2.08 | 18.97 ± 1.33 | -1.906 | 0.067 | 0.119 |
| Tapping (number of taps/ 10 sec.) |  |  |  |  |  |
| PRE | 85.52 ± 15.20 | 79.80 ± 7.69 | 0.811 | 0.425 | 0.025 |
| POST | 87.65 ± 13.83 | 80.80 ± 4.76 | 1.080 | 0.290 | 0.043 |
| PVT (mean reaction time ms) |  |  |  |  |  |
| PRE | 319.32 ± 26.99 | 316.93 ± 19.86 | 0.186 | 0.854 | 0.001 |
| POST | 323.11 ± 28.35 | 323.54 ± 40.11 | -0.029 | 0.977 | < 0.001 |
| IQ (Standard progressive matrices) | 99.88 ± 11.94 | 100.40 ± 10.50 | -0.091 | 0.928 | <0.001 |

**Table 2.** Behavioral measures male vs. female within SLEEP (n=15; 13 male) and WAKE (n=14; 11 male) group.

| BEHAVIORAL MEASURES | | | | | | | | | | |
| --- | --- | --- | --- | --- | --- | --- | --- | --- | --- | --- |
|  | WAKE | | | | | SLEEP | | | | |
|  | male | female | t | P | η^2^ | male | female | t | P | η^2^ |
| SDSA [°] |  |  |  |  |  |  |  |  |  |  |
| PRE (last 2 training blocks) | 21.43 ± 2.60 | 23.57 ± 2.19 | -1.298 | 0.219 | 0.123 | 21.42 ± 1.86 | 21.26 ± 2.88 | 0.107 | 0.917 | 0.001 |
| POST (2 retrieval blocks) | 21.36 ± 2.17 | 23.53 ± 1.75 | -1.582 | 0.140 | 0.173 | 21.21 ± 2.64 | 22.41 ± 3.15 | -0.588 | 0.566 | 0.026 |
| Riding time [s] |  |  |  |  |  |  |  |  |  |  |
| PRE (last 2 training blocks) | 20.00 ± 2.36 | 21.92 ± 0.67 | -1.357 | 0.200 | 0.133 | 19.27 ± 2.64 | 19.83 ± 1.91 | -0.287 | 0.779 | 0.006 |
| POST (2 retrieval blocks) | 17.63 ± 2.32 | 19.28 ± 1.21 | -1.163 | 0.267 | 0.101 | 16.66 ± 1.83 | 18.50 ± 1.84 | -1.323 | 0.209 | 0.119 |
| Tapping (number of taps/ 10 sec.) |  |  |  |  |  |  |  |  |  |  |
| PRE | 83.09 ± 17.92 | 75.00 ± 2.65 | 0.758 | 0.463 | 0.046 | 87.78 ± 12.59 | 87.00 ± 7.07 | 0.080 | 0.937 | 0.001 |
| POST | 86.00 ± 15.25 | 80.00 ± 2.65 | 0.660 | 0.522 | 0.035 | 89.17 ± 12.88 | 82.00 ± 8.49 | 0.747 | 0.470 | 0.044 |
| PVT (mean reaction time ms) |  |  |  |  |  |  |  |  |  |  |
| PRE | 321.63 ± 25.37 | 316.61 ± 26.05 | 0.303 | 0.767 | 0.008 | 317.21 ± 29.36 | 317.43 ± 14.81 | -0.010 | 0.992 | < 0.001 |
| POST | 325.55 ± 27.47 | 333.61 ± 53.18 | -0.373 | 0.716 | 0.011 | 321.04 ± 30.03 | 308.43 ± 4.24 | 0.575 | 0.575 | 0.025 |
| IQ (Standard progressive matrices) | 96.55 ± 11.18 | 104.00 ± 12.12 | -1.009 | 0.333 | 0.078 | 102.69 ± 12.26 | 95.00 ± 7.071 | 0.848 | 0.412 | 0.052 |

**Table 3.** Sleep architecture male (n=13) vs. female (n=2).

| SLEEP ARCHITECTURE | | | | | | | | | | | | | | | |
| --- | --- | --- | --- | --- | --- | --- | --- | --- | --- | --- | --- | --- | --- | --- | --- |
|  | Adaptation | | | | | Learning | | | | | Control | | | | |
|  | male | female | t | P | η^2^ | male | female | t | P | η^2^ | male | female | t | P | η^2^ |
|  |  |  |  |  |  |  |  |  |  |  |  |  |  |  |  |
| TST | 510.19 ± 12.09 | 510.25 ± 5.30 | -0.006 | 0.995 | < 0.001 | 505.77 ± 18.33 | 491.75 ± 19.45 | 1.143 | 0.335 | 0.072 | 499.65 ± 9.48 | 495.00 ± 12.02 | 0.322 | 0.752 | 0.008 |
| SOL | 17.81 ± 10.28 | 20.00 ± 13.44 | -0.274 | 0.789 | 0.006 | 17.96 ± 13.45 | 13.25 ± 5.30 | 1.002 | 0.641 | 0.017 | 16.85 ± 8.80 | 4.75 ± 6.01 | 1.847 | 0.088 | 0.208 |
| EFF | 96.87 ± 0.97 | 96.33 ± 1.97 | 0.663 | 0.519 | 0.033 | 97.15 ± 2.48 | 97.84 ± 1.53 | 0.477 | 0.714 | 0.011 | 97.15 ± 2.17 | 96.79 ± 2.40 | 0.221 | 0.828 | 0.004 |
| WASO | 7.88 ± 4.65 | 4.00 ± 0.71 | 1.143 | 0.273 | 0.091 | 6.50 ± 4.27 | 6.75 ± 7.42 | -0.072 | 0.944 | < 0.001 | 7.23 ± 6.79 | 12.25 ± 6.72 | -0.974 | 0.348 | 0.068 |
| N1 % | 6.08 ± 1.70 | 6.61 ± 0.55 | -0.424 | 0.678 | 0.014 | 5.70 ± 2.70 | 5.83 ± 1.28 | -0.061 | 0.952 | < 0.001 | 6.05 ± 1.48 | 8.15 ± 1.27 | -1.883 | 0.082 | 0.214 |
| N2 % | 38.66 ± 5.99 | 44.55 ± 7.16 | -1.273 | 0.225 | 0.111 | 39.83 ± 8.25 | 46.21 ± 5.58 | -1.039 | 0.318 | 0.077 | 39.20 ± 6.81 | 40.83 ± 9.99 | -0.301 | 0.768 | 0.007 |
| N3 % | 31.42 ± 6.02 | 28.55 ± 5.28 | 0.635 | 0.536 | 0.030 | 31.7 ± 6.45 | 29.55 ± 8.22 | 0.443 | 0.665 | 0.015 | 32.79 ± 6.98 | 30.86 ± 8.61 | 0.358 | 0.726 | 0.010 |
| REM % | 23.83 ± 3.40 | 20.30 ± 2.43 | 1.394 | 0.187 | 0.130 | 22.69 ± 8.29 | 18.43 ± 1.36 | 0.703 | 0.494 | 0.037 | 21.97 ± 3.53 | 20.17 ± 2.66 | 0.681 | 0.508 | 0.034 |
| SPINDLE ACITVITY (SPA) | | | | | | | | | | | | | | | |
|  | Adaptation | | | | | Learning | | | | | Control | | | | |
|  | male | female | t | P | η^2^ | male | female | t | P | η^2^ | male | female | t | P | η^2^ |
|  |  |  |  |  |  |  |  |  |  |  |  |  |  |  |  |
| N2  LEFT | 19.29 ± 2.62 | 22.01 ± 0.01 | -1.424 | 0.180 | 0.145 | 19.13 ± 3.00 | 20.74 ± 2.55 | -0.710 | 0.491 | 0.040 | 19.17 ± 3.04 | 22.21 ± 2.98 | -1.315 | 0.213 | 0.126 |
| N2 RIGHT | 19.47 ± 3.35 | 20.42 ± 0.42 | -0.387 | 0.706 | 0.012 | 18.99 ± 3.27 | 21.15 ± 2.18 | -0.884 | 0.394 | 0.061 | 18.78 ± 1.69 | 22.17 ± 0.33 | -2,740 | **0.018** | 0.385 |
| REM DURATION MEASURES | | | | | | | | | | | | | | | |
|  | Adaptation | | | | | Learning | | | | | Control | | | | |
|  | male | female | t | P | η^2^ | male | female | t | P | η^2^ | male | female | t | P | η^2^ |
|  |  |  |  |  |  |  |  |  |  |  |  |  |  |  |  |
| REM | 131.69 ± 19.67 | 105.00 ± 9.19 | 1.843 | 0.088 | 0.207 | 122.38 ± 48.91 | 90.00 ± 9.19 | 0.906 | 0.381 | 0.059 | 119.92 ± 23.29 | 107.25 ± 10.25 | 0.740 | 0.472 | 0.040 |
| Tonic | 59.35 ± 15.92 | 59.25 ± 10.25 | 0.008 | 0.994 | < 0.001 | 55.96 ± 25.07 | 42.50 ± 6.36 | 0.517 | 0.614 | 0.020 | 55.69 ± 14.04 | 63.50 ± 22.63 | -0.691 | 0.502 | 0.035 |
| Phasic | 62.15 ± 11.10 | 44.2 ± 1.06 | 2.210 | **0.046** | 0.273 | 59.04 ± 28.60 | 48.25 ± 3.89 | 0.734 | 0.476 | 0.040 | 53.88 ± 11.58 | 36.50 ± 7.07 | 2.027 | 0.064 | 0.240 |

TST: Total sleep time (min.); SOL: Sleep onset latency (min.); EFF: Sleep efficiency (%); WASO: Wake after sleep onset (min.)
